# Commentary: Pre-crastination: hastening subgoal completion at the expense of extra physical effort

**DOI:** 10.3389/fpsyg.2015.01269

**Published:** 2015-08-24

**Authors:** Michael Richter

**Affiliations:** School of Natural Sciences and Psychology, Liverpool John Moores UniversityLiverpool, UK

**Keywords:** pre-crastination, effort, law of least effort, choice, reward

Rosenbaum et al. (2014) announced in a recent *Psychological Science* paper the discovery of a new psychological phenomenon. They presented nine studies on task choice in a bucket carrying task and claimed that the results of these studies provide evidence for pre-crastination, the tendency to complete (sub)tasks as soon as possible, even if this comes with the expense of additional physical effort. In this commentary, I discuss whether the findings of Rosenbaum and colleagues indeed reveal a new, surprising phenomenon or are old wine in new skins.

Participants in Rosenbaum and colleagues' studies had to choose one of two buckets and to carry the selected bucket to a target position. Systematically varying the distance between participants and buckets as well as the distance between buckets and target position, Rosenbaum and colleagues observed that participants frequently chose the bucket that was closer to them, even if this implied a longer carrying distance and thus a higher total physical effort. They were surprised by this finding and explained it by participants' motivation to complete the first subtask (choosing a bucket) as soon as possible. They suggested that (task) goals impose a memory load and that individuals' primary motivation is to free the memory from this load by attaining the goal.

I agree that the finding that individuals are more interested in freeing their memory from the load imposed by a goal than in minimizing energy investment would constitute a new phenomenon. However, I doubt that Rosenbaum and colleagues' studies provide evidence that is strong enough to warrant such a claim. The claim about the discovery of a new phenomenon is only warranted if the empirical findings are indeed new and have not been described before and if the findings cannot be explained by existing theoretical approaches. I will show in the following that both premises do not hold.

First, there is already empirical research that has demonstrated that, under certain conditions, individuals prefer effortful tasks over effortless tasks. For instance, Eisenberger et al. (1985) demonstrated that children who could choose between earning 2 cents for doing nothing and 3 cents for copying nonsense words chose more often the higher reward even if this implied a higher effort. This empirical finding fits well with Rosenbaum and colleagues' suggestion that keeping busy is more rewarding then doing nothing and that participants might have preferred the closer bucket because it was the more attractive, rewarding option. Buckert et al.'s study (1979) on achievement motivation constitutes a second example of research that revealed humans' preference for effortful tasks. They observed that participants preferred task items that allowed them to acquire information about their own ability even if these items required more effort than less diagnostic items.

Second, there are a handful of theoretical approaches that offer explanations why individuals sometimes prefer effortful tasks. Nicholls (1984) suggested that individuals are primarily interested in demonstrating high ability and in avoiding demonstrating low ability when performing achievement tasks. He reasoned that easy tasks that require a low amount of effort do not allow the demonstration of a high ability because success is certain and everyone succeeds. In contrast, succeeding in difficult tasks that require a moderate amount of effort is more informative because only individuals with a high ability will be able to succeed. According to Nicholls' analysis, individuals should consequently prefer tasks that require a moderate amount of effort over tasks that require low effort. Eisenberger's (1992) secondary reward theory of industriousness constitutes another example of a theoretical approach that predicts that individuals prefer effortful tasks. He suggested that effort can become a secondary reinforcer when contingent rewards are repeatedly experienced when exerting effort. Individuals will then prefer tasks that require moderate effort over easy, effortless tasks because the effort associated with the moderately difficulty tasks signals potential rewards. Classical approaches that assume that effort mobilization is governed by energy conservation concerns—like the law of least effort (e.g, Tolman, 1932; Hull, 1943; Zipf, 1949) or motivational intensity theory (Brehm and Self, 1989)—might also offer an explanation for Rosenbaum and colleagues' findings. If participants followed Rosenbaum and colleagues' instructions to choose the easiest task, their results might indicate that it felt less demanding to quickly start the carrying task and to carry the bucket a long way than to start the carrying task later and to carry the bucket a short way. If this holds, Rosenbaum and colleagues' findings are not new but a mere replication of preceding studies on the law of least effort. Participants chose among two options the option that appeared to be the less demanding one.

The cited empirical evidence and theoretical accounts question Rosenbaum and colleagues' claim about the discovery of a new phenomenon. The observation that individuals prefer effortful tasks is not new and there are theoretical approaches that can account for Rosenbaum and colleagues' findings. I do not intend to suggest that the pre-crastination explanation is wrong. Rosenbaum and colleagues might have discovered a new phenomenon. Participants in their studies might indeed have been more concerned with completing the first subtasks to free their memory from the load that the subgoal imposed than with minimizing effort. However, given that the presented data also fit with preceding research and theorizing, the strong claim about the discovery of a new phenomenon seems premature. Rosenbaum and colleagues' studies demonstrated that individuals sometimes prefer the (physically) more effortful task but they did not provide information regarding the underlying motivation or mechanisms. Participants might have been motivated to complete the first subtask. However, they might also have chosen the physically more effortful task because it allowed the demonstration of high ability, because of learned industriousness, because it was more attractive, or because of the lower overall demand associated with this option. It is essential to rule out these possible alternative explanations before the claim of the discovery of a new phenomenon is warranted.

## Conflict of interest statement

The author declares that the research was conducted in the absence of any commercial or financial relationships that could be construed as a potential conflict of interest.
